# Flux balance analysis predicts Warburg-like effects of mouse hepatocyte deficient in miR-122a

**DOI:** 10.1371/journal.pcbi.1005618

**Published:** 2017-07-07

**Authors:** Hua-Qing Wu, Mei-Ling Cheng, Jin-Mei Lai, Hsuan-Hui Wu, Meng-Chun Chen, Wen-Huan Liu, Wu-Hsiung Wu, Peter Mu-Hsin Chang, Chi-Ying F. Huang, Ann-Ping Tsou, Ming-Shi Shiao, Feng-Sheng Wang

**Affiliations:** 1 Department of Chemical Engineering, National Chung Cheng University, Chiayi, Taiwan; 2 Department of Biomedical Sciences, Chang Gung University, Tao-Yuan, Taiwan; 3 Metabolomics Core Laboratory, Healthy Aging Research Center, Chang Gung University, Tao-Yuan, Taiwan; 4 Clinical Phenome Center, Chang Gung Memorial Hospital, Linkou, Tao-Yuan, Taiwan; 5 Department of Life Science, Fu-Jen Catholic University, New Taipei City, Taiwan; 6 Institute of Biopharmaceutical Sciences, National Yang-Ming University, Taipei, Taiwan; 7 Department of Oncology, Taipei Veterans General Hospital, Taipei, Taiwan; 8 Faculty of Medicine, National Yang Ming University, Taipei, Taiwan; 9 Department of Biotechnology and Laboratory Science in Medicine, National Yang-Ming University, Taipei, Taiwan; 10 Department of Biochemistry, College of Medicine, Kaohsiung Medical University, Kaohsiung, Taiwan; Chalmers University of Technology, SWEDEN

## Abstract

The liver is a vital organ involving in various major metabolic functions in human body. MicroRNA-122 (miR-122) plays an important role in the regulation of liver metabolism, but its intrinsic physiological functions require further clarification. This study integrated the genome-scale metabolic model of hepatocytes and mouse experimental data with germline deletion of *Mir122a* (*Mir122a*^*–/–*^) to infer Warburg-like effects. Elevated expression of *MiR-122a* target genes in *Mir122a*^*–/–*^mice, especially those encoding for metabolic enzymes, was applied to analyze the flux distributions of the genome-scale metabolic model in normal and deficient states. By definition of the similarity ratio, we compared the flux fold change of the genome-scale metabolic model computational results and metabolomic profiling data measured through a liquid-chromatography with mass spectrometer, respectively, for hepatocytes of 2-month-old mice in normal and deficient states. The *Ddc* gene demonstrated the highest similarity ratio of 95% to the biological hypothesis of the Warburg effect, and similarity of 75% to the experimental observation. We also used 2, 6, and 11 months of mir-122 knockout mice liver cell to examined the expression pattern of DDC in the knockout mice livers to show upregulated profiles of DDC from the data. Furthermore, through a bioinformatics (LINCS program) prediction, BTK inhibitors and withaferin A could downregulate DDC expression, suggesting that such drugs could potentially alter the early events of metabolomics of liver cancer cells.

## Author summary

For almost a century, researchers have known that cancer cells have an abnormal metabolism and utilize glucose differently than normal cells do. Aerobic glycolysis or the Warburg effect in cancer cells involves elevated glucose uptake with lactic acid production in the presence of oxygen. MicroRNAs have recently been discovered to be key metabolic regulators that mediate the fine tuning of genes that are involved directly or indirectly in cancer metabolism. MicroRNA-122 (miR-122) plays an important role in the regulation of liver metabolism, but its intrinsic physiological functions require further clarification. This study integrated the genome-scale metabolic modeling (GSMM) of hepatocytes and mouse experimental data with germline deletion of *Mir122a* (*Mir122a*^*–/–*^) to infer Warburg-like effects. *In silico* and *in vivo* observations indicated that DDC overexpression induced Warburg effect in hepatocyte. Furthermore, through a bioinformatics prediction, BTK inhibitors and withaferin A could downregulate DDC expression, suggesting that such drugs could potentially alter the early events of metabolomics of liver cancer cells.

## Introduction

Cancer cell metabolism is an exciting field of biology that provides a novel approach for treating cancer [1–8]. For almost a century, researchers have known that cancer cells have an abnormal metabolism and utilize glucose differently than normal cells do. However, glucose uptake may reveal only part of a cancer’s metabolic system [1–8]. Cancer cells have become habituated to certain fuel sources and metabolic pathways (“metabolic reprogramming”), profoundly changing how they consume and utilize nutrients such as glucose. Inhibiting key enzymes in these metabolic pathways can disrupt tumor cell proliferation and survival without affecting normal cells. The metabolic reprogramming of cancer cells is also linked to specific genetic alterations in oncogenes and tumor suppressor genes. Hence, a systems biology approach, which involves integrating genetic, protein-protein interaction and metabolic networks, may be a useful tool for discovering and developing novel targeted cancer therapeutics.

A superior understanding of the genome-scale human metabolic network may lead to the identification of disease genes and related pathways, which may be more appropriate targets for drug development. The development of genome-scale human metabolic networks, such as Recon 1 and 2 [9, 10], the Edinburgh human metabolic network (EHMN) [11], and human metabolic reactions [12, 13], has resulted in the emergence of network medicine. Network medicine aims to understand the structure and function of the human genome and to provide a connection between the genotype and phenotype [14]. Human metabolism is complex and very specialized in different tissues and cell types. Studies of the human metabolism have focused on reconstructing tissue-specific metabolic networks [13, 15, 16]. These previously mentioned genome-scale reconstructions of the human metabolic network are an excellent basis for reconstructing tissue-specific metabolic networks. HepatoNet1, the first manually reconstructed tissue-specific network of human hepatocytes, was assembled according to two global reconstructions, Recon1 and EHMN, and metabolic pathways in the *Kyoto Encyclopedia of Genes and Genomes* (KEGG) [15]. This reconstructed network consists of 777 metabolites in eight compartments (six intracellular and two extracellular) and 2539 reactions, including 1466 transport reactions. The network was curated using more than 1500 primary articles, reviews, and biochemical textbooks. Recently, many algorithms, including the Model Building Algorithm (MBA) [17] and the metabolic Context-specificity Assessed by Deterministic Reaction Evaluation (mCADRE) method [18], have been proposed for inferring tissue-specific subnetworks from generic genome-scale human metabolic networks. Two liver-specific metabolic networks, liverMBA and liverCADRE, generated using MBA and mCADRE, respectively, have been used to predict potential drug targets and improve metabolic flux predictions [19, 20]. The developers of mCADRE claimed that liverCADRE exhibited similar or more improved coverage and higher functionality than the existing models. In addition to these two liver-specific metabolic networks for the normal liver, MBA and mCADRE have been used separately to generate metabolic networks for liver cancer.

MicroRNAs have recently been discovered to be key metabolic regulators that mediate the fine tuning of genes that are involved directly or indirectly in cancer metabolism [21]. Mouse studies have revealed that microRNA-122 (miR-122), which accounts for 70% of the total miRNAs in the liver, plays a pivotal role in liver and has been implicated as a regulator of fatty acid metabolism. Reduced miR-122 levels are associated with hepatocellular carcinoma (HCC), and miR-122 plays a crucial positive role in the regulating hepatitis C virus replication [22]. However, the intrinsic physiological roles of miR-122 remain largely undetermined. Tsai et al. demonstrated that mice lacking the gene encoding miR-122a (*Mir122a*^*–/–*^) (hereafter referred to as *Mir122a*^*–/–*^mice) are viable but developed temporally controlled steatohepatitis, fibrosis, and HCC [23]. However, how miR-122 affects the metabolic network of hepatocytes is unclear. This study aimed to reveal this metabolic reprogramming mechanism by integrating the flux balance analysis (FBA) of a genome-scale metabolic model of hepatocytes and the experimental data of *Mir122a*^*–/–*^mice. Several new targets and inhibitors, which could modulate the Warburg effect, are emerged from this integrated metabolomic analysis and warrant further investigation in a future clinical study.

## Results

### Metabolomic analyses of miR-122a deficient mice

**Fig 1:**
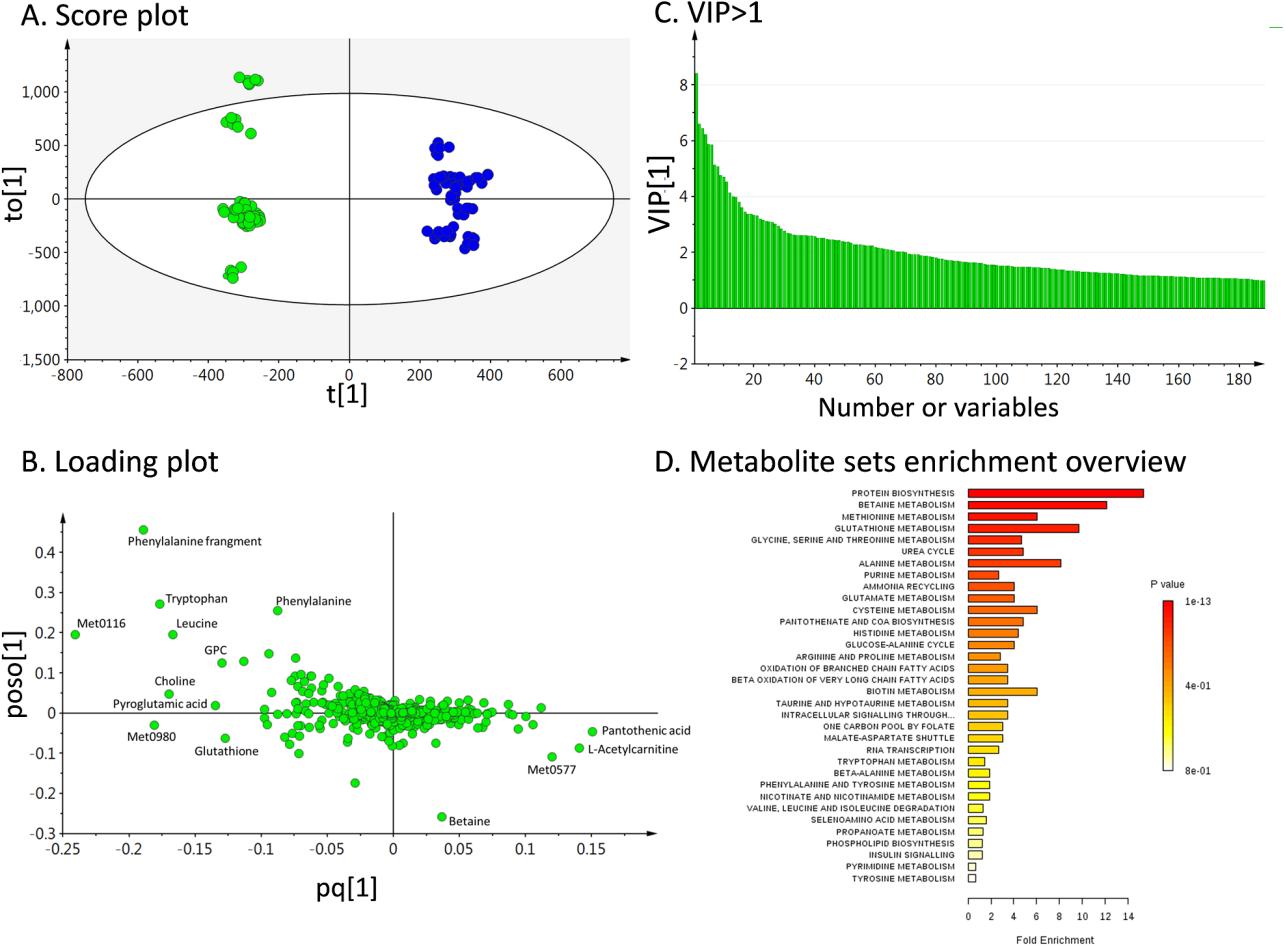
Metabolomic data in miR-122a deficient mice. (A) Orthogonal partial least-squares discriminant analysis (OPLS-DA) score plot of metabolite profiles derived from 10 miR-122a deficient mice (green) and the 10 control mice (blue) with corresponding loading plot. The ellipse shown in the model represents the Hotelling T2 with 95% confidence. Each data point represents one mouse liver sample, and the distance between points in the plot indicates the similarity between samples. (B) OPLS-DA loading plot of liver metabolite profiles, each point in represents one feature (or metabolite). (C) Plot of variables importance for the projection (VIP) summarizes the importance of the variables (Fig 1A and 1B). The VIP ranking priority was according to the VIP values, and metabolites with VIP >1 are shown. (D) The 35 metabolites were used for metabolite set enrichment analysis (MSEA). These metabolite sets are ranked according to the Holm P value with hatched lines shown. Liver tissue samples were extracted by the Folch method, and the aqueous (upper) phases were analyzed using LC-TOFMS in the electrospray positive ion mode. Each sample analysis consists of six replicates. A web-based tool (www.metaboanalyst.ca) for metabolite set enrichment analysis was used for the analysis. Detail metabolomics differences were revealed by OPLS-DA model (R^2^X = 0.612, Q2 = 0.984). The axes, t[1], to[1], pq[1], and poso[1] depict the predictive component, the first orthogonal component, the predictive component loadings, and the first orthogonal loadings, respectively, of the OPLS-DA model.

For the untargeted metabolomic analysis, 20 liver tissue samples including 10 control mice and 10 *Mir122a*^*–/–*^mice were extracted using the Folch method, and the aqueous phases were analyzed by LC-TOFMS in the electrospray positive-ion mode (S1 Fig). In the metabolomic profiling, 1234 positive-mode features were identified and applied for SIMCA-P analysis. The orthogonal partial least squares discriminant analysis (OPLS-DA) score plot and loading plot showed remarkable separation between the controls and *Mir122a*^*–/–*^mice (Fig 1A and 1B). The variable importance in the projection (VIP) values of those variables greater than 1.0 is shown in Fig 1C and Table 1. Thirty-five metabolites with VIP values > 1.0 were included in the metabolite set enrichment analysis (MSEA). The datasets were also analyzed using the Metaboanalyst platform. Fig 1D shows the metabolome view of the affected pathways. The results of the untargeted analysis revealed these metabolites to be important discriminators of the healthy controls and *Mir122a*^*–/–*^mice.

**Table 1:** The fold changes of the 35 intracellular metabolites from the LC/MS analysis and 16 miR122a target enzyme computational predictions.

Metabolite	Compartment	Exp (D/N)	DDC	PKM	UROD	CPOX	HMBS	FECH	GPX7	TXNRD1	SPTLC1	SCD	RPIA	GYS1	ALPL	ASAH1	SGPL1	PAFAH1B1
Leucine	c, m	**↑**	**↑**	**↑**	**↑**	**↑**	**↑**	**↑**	**↑**	**↑**	**↑**	**↑**	**↑**	**↓**	**↓**	**↓**	**↓**	**↑**
Choline	c, n	**↑**	**↑**	**↓**	**↑**	**↑**	**↑**	**↑**	**↑**	**↑**	**↑**	**↓**	**↑**	**↑**	**↑**	**↑**	**↓**	**↓**
Pantothenic acid	c	**↓**	**↓**	**↑**	**↑**	**↑**	**↑**	**↑**	**↑**	**↑**	**↑**	**↑**	**↑**	**↓**	**↓**	**↑**	**↑**	**↑**
L-Acetylcarnitine	c, m, x	**↓**	**↑**	**↑**	**↑**	**↑**	**↑**	**↑**	**↓**	**↑**	**↑**	**↑**	**↑**	**↑**	**↑**	**↑**	**↑**	**↑**
Glutathione	c, m	**↑**	**↑**	**↑**	**↑**	**↑**	**↑**	**↑**	**↑**	**↑**	**↓**	**↑**	**↓**	**↓**	**↑**	**↓**	**↓**	**↓**
L-Tryptophan	c	**↑**	**↑**	**↑**	**↑**	**↑**	**↑**	**↑**	**↑**	**↑**	**↑**	**↑**	**↑**	**↓**	**↓**	**↑**	**↑**	**↑**
L-Glutamine	c, m	**↑**	**↑**	**↑**	**↑**	**↑**	**↑**	**↑**	**↑**	**↑**	**↓**	**↑**	**↑**	**↑**	**↓**	**↓**	**↓**	**↑**
S-Formylglutathione	c	**↑**	**↑**	**↑**	**↑**	**↑**	**↑**	**↑**	**↑**	**↑**	**↑**	**↑**	**↑**	**↑**	**↑**	**↑**	**↑**	**↑**
L-Cysteine	c	**↑**	**↑**	**↑**	**↑**	**↑**	**↑**	**↑**	**↑**	**↑**	**↑**	**↑**	**↑**	**↓**	**↓**	**↓**	**↓**	**↑**
Phenylalanine	c	**↑**	**↑**	**↑**	**↑**	**↑**	**↑**	**↑**	**↑**	**↑**	**↑**	**↑**	**↑**	**↑**	**↓**	**↑**	**↑**	**↑**
L-Proline	c, m	**↑**	**↑**	**↑**	**↑**	**↑**	**↑**	**↑**	**↑**	**↓**	**↑**	**↑**	**↑**	**↑**	**↓**	**↓**	**↓**	**↓**
Hydroxybutyrylcarnitine	c	**↓**	**↓**	**↓**	**↓**	**↓**	**↓**	**↓**	**↓**	**↑**	**↓**	**↓**	**↓**	**↑**	**↑**	**↓**	**↓**	**↑**
L-Alanine	c, m, x	**↑**	**↑**	**↑**	**↑**	**↑**	**↑**	**↑**	**↑**	**↑**	**↑**	**↓**	**↑**	**↑**	**↓**	**↑**	**↑**	**↑**
AMP	c, m, r, x	**↑**	**↓**	**↑**	**↑**	**↑**	**↑**	**↑**	**↓**	**↑**	**↓**	**↓**	**↓**	**↓**	**↑**	**↓**	**↑**	**↓**
Creatine	c	**↑**	**↑**	**↓**	**↑**	**↓**	**↓**	**↓**	**↑**	**↑**	**↓**	**↑**	**↑**	**↓**	**↑**	**↓**	**↑**	**↓**
Adenine	c	**↑**	**↑**	**↑**	**↑**	**↑**	**↑**	**↑**	**↑**	**↑**	**↑**	**↓**	**↑**	**↓**	**↓**	**↑**	**↓**	**↑**
L-Methionine	c	**↑**	**↑**	**↑**	**↑**	**↑**	**↑**	**↑**	**↑**	**↑**	**↑**	**↑**	**↑**	**↓**	**↓**	**↑**	**↓**	**↑**
Biotin	c, m, n	**↓**	**↓**	**↓**	**↓**	**↓**	**↓**	**↓**	**↓**	**↑**	**↓**	**↓**	**↓**	**↓**	**↓**	**↓**	**↓**	**↓**
Cysteinylglycine	c	**↑**	**↑**	**↑**	**↑**	**↑**	**↑**	**↑**	**↑**	**↑**	**↓**	**↓**	**↓**	**↓**	**↑**	**↓**	**↓**	**↑**
L-Glutamic acid	c, m	**↑**	**↑**	**↑**	**↑**	**↑**	**↑**	**↑**	**↑**	**↑**	**↑**	**↑**	**↑**	**↓**	**↑**	**↓**	**↑**	**↓**
L-Valine	c, m	**↑**	**↑**	**↓**	**↑**	**↑**	**↑**	**↑**	**↑**	**↑**	**↑**	**↑**	**↑**	**↓**	**↓**	**↑**	**↑**	**↑**
L-Carnitine	c, m, x	**↓**	**↑**	**↑**	**↑**	**↑**	**↑**	**↑**	**↑**	**↑**	**↓**	**↑**	**↑**	**↓**	**↓**	**↑**	**↑**	**↑**
Dimethylglycine	c, m	**↑**	**↑**	**↓**	**↑**	**↑**	**↑**	**↑**	**↑**	**↑**	**↑**	**↓**	**↑**	**↓**	**↓**	**↑**	**↓**	**↑**
L-Histidine	c, m	**↑**	**↑**	**↑**	**↑**	**↑**	**↑**	**↑**	**↑**	**↓**	**↑**	**↑**	**↑**	**↑**	**↑**	**↑**	**↑**	**↑**
Adenosine	c, m	**↑**	**↑**	**↑**	**↑**	**↑**	**↑**	**↑**	**↑**	**↑**	**↑**	**↓**	**↑**	**↓**	**↓**	**↑**	**↓**	**↑**
Valerylcarnitine	c	**↓**	**↓**	**↑**	**↓**	**↓**	**↓**	**↓**	**↓**	**↑**	**↑**	**↓**	**↑**	**↑**	**↓**	**↑**	**↓**	**↑**
Betaine	c	**↓**	**↑**	**↓**	**↑**	**↑**	**↑**	**↑**	**↑**	**↑**	**↑**	**↓**	**↑**	**↓**	**↓**	**↑**	**↓**	**↑**
Hexanoylcarnitine	c, x	**↓**	**-**	**-**	**-**	**-**	**-**	**-**	**-**	**-**	**↑**	**-**	**-**	**-**	**-**	**-**	**-**	**-**
5-Methyltetrahydrofolic acid	c	**↓**	**↑**	**↑**	**↓**	**↑**	**↑**	**↑**	**↑**	**↓**	**↑**	**↓**	**↑**	**↑**	**↓**	**↑**	**↓**	**↑**
Niacinamide	c	**↓**	**-**	**-**	**↑**	**-**	**-**	**-**	**-**	**-**	**-**	**-**	**↑**	**-**	**-**	**-**	**-**	**↑**
L-Threonine	c	**↑**	**↑**	**↑**	**↑**	**↑**	**↑**	**↑**	**↑**	**↑**	**↑**	**↑**	**↑**	**↓**	**↓**	**↑**	**↑**	**↑**
2-Aminomuconic semialdehyde	c	**↓**	**-**	**-**	**-**	**-**	**-**	**-**	**-**	**↑**	**↑**	**↑**	**↑**	**↑**	**↑**	**-**	**-**	**-**
Homovanillic acid	m	**↓**	**↑**	**↑**	**↑**	**↑**	**↑**	**↑**	**↑**	**↑**	**↑**	**↑**	**↑**	**↑**	**↑**	**↑**	**↑**	**↑**
Cholesterol*	c, l, m, r	**↑**	**↑**	**↓**	**↑**	**↑**	**↑**	**↑**	**↑**	**↓**	**↑**	**↑**	**↑**	**↓**	**↓**	**↓**	**↑**	**↓**
Guanine	c	**↑**	**↑**	**↓**	**↑**	**↑**	**↑**	**↑**	**↑**	**↑**	**↑**	**↓**	**↑**	**↑**	**↑**	**↓**	**↑**	**↓**
Similarity ratio			0.75	0.556	0.694	0.639	0.639	0.639	0.5	0.556	0.583	0.583	0.444	0.417	0.361	0.417	0.417	0.472

D/N ratio is the fold change of metabolic concentration or flux in the dysregulated state (D) and normal state (N), which are indicated as “**↑**”(increase), “**↓**”(decrease), and “-”(no change). (Please see the S1 Table for the 20 miR122a target enzymes computational prediction). The fold change (FC) is computed using Eq (6). The positive/negative value of the logarithmic FC indicates the increase or decrease.

* cholesterol was analyzed by NMR. Symbol of compartment: c (cytoplasm), l (lysosome), m (mitochondrion), n (nucleus), r (endoplasmic reticulum), and x (peroxisome)

### Similarity effect as compared with the Warburg effect hypothesis and metabolomics profiling data of miR-122a deficient mice

A tissue-specific metabolic model of hepatocytes obtained from the supplementary data of Recon 2 and cell type-specific models [10], hereafter referred to as the Recon 2-hepatocyte model, was applied to evaluate flux distributions under normal or miR-122 dysregulated conditions. In this study, the maximization of the ATP production rate was considered the cellular objective.

According to the physiological data of mice reported by Trotman et al. [24], we restricted the secretion rates of direct bilirubin and indirect bilirubin to 1.7 ≤ *v*_direct_bilirubin_≤ 8.55 μmol/L/day and 0 ≤ *v*_indirect_bilirubin_≤ 6.84 μmol/L/day, respectively. A minimal medium containing the selected nutrients of glucose, ammonia, sulfate, and phosphate was commonly used to predict cell growth in the study of FBA. This work uses the data of *Mir122a* knockout mice from Tsai et al. [23, 25]. All the mice used in this study were male mice of 2-month old. The wild-type and knockout mice are fed by the Laboratory Autoclavable Rodent Diet 5010, which the ingredients are described in Materials and Methods.

Tsai et al. [23, 25] applied a miRNA-target interaction database to predict miR-122 target genes in mice and humans (S2 and S3 Tables). In this study, using the KEGG and ExPASy databases, we determined that 20 genes from the set of miR-122 target genes directly encode enzymes listed in the Recon2-hepatocyte model. The target genes and their regulated reactions are shown in S4 Table. The elevated expressions of the 20 target genes induced by *Mir122a* deletion were applied to modulate the flux distributions in the normal and deficient states (S2 Fig).

**Fig 2:**
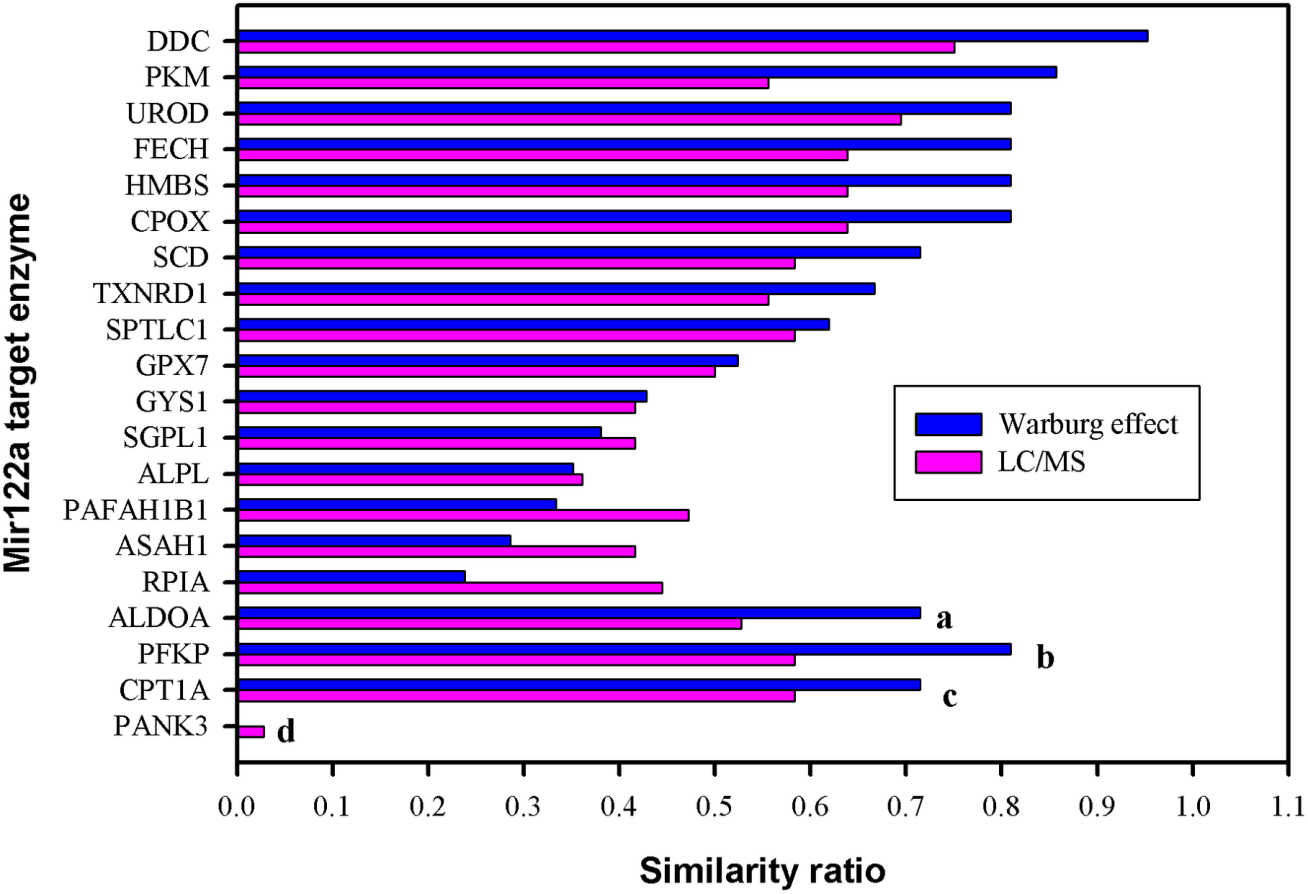
Similarity ratio of 20 miR-122a target enzymes to Warburg effect and experimental metabolic profiling observations (LC/MS). The similarity ratio of each target gene was calculated to indicate that how many percentages for the computational predictions (100% overexpression) are similar to the hypothesis of the Warburg effect (blue line) and experimental metabolic profiling observations (pink line). The Ddc gene received the highest similarity ratio to the Warburg effect (0.952) and experiments (0.75) (a, 54% overexpression; b, downregulation; c, knockout; d, overexpression of forward and reverse reactions).

We detected a group of 35 metabolites with significant variable importance for projection (VIP) scores > 1 (S5 Table) in the liquid chromatography–mass spectrometry (LC/MS) analysis. Of these, 12 metabolites had a decreased mass-to-charge ratio (m/z), and 23 metabolites had an increased m/z ratio. Table 1 shows the similarity effect of the computational prediction for each miR-122 target gene compared with the data for the 35 metabolites. We observed that *Ddc*, the gene encoding 3,4-dihydroxy-L-phenylalanine (L-DOPA) decarboxylase (DDC), exhibited the highest similarity ratio to the Warburg effect hypothesis (0.952, S5 Table listed metabolites used for evaluation), and the experimental metabolomics profiling data (0.75), as shown in Fig 2. DDC, which is widely distributed throughout the body, is a pyridoxal-phosphate (PLP)-dependent enzyme that catalyzes L-DOPA to dopamine and 5-hydroxy-Ltryptophan (5-HTP) to serotonin [26–28]. DDC primarily participates in the synthesis of amines that are involved in angiogenesis, cell proliferation, and differentiation [29, 30]. Elevated DDC expression has been considered a potential novel biomarker for various cancer types, including neuroendocrine malignancies [31–33], small-cell lung carcinoma [34, 35], neuroblastoma [36], prostate cancer [37], colorectal adenocarcinoma [38] and laryngeal cancer[39]. In this study, we investigated the role DDC in the metabolic reprogramming of cancer cells.

**Fig 3:**
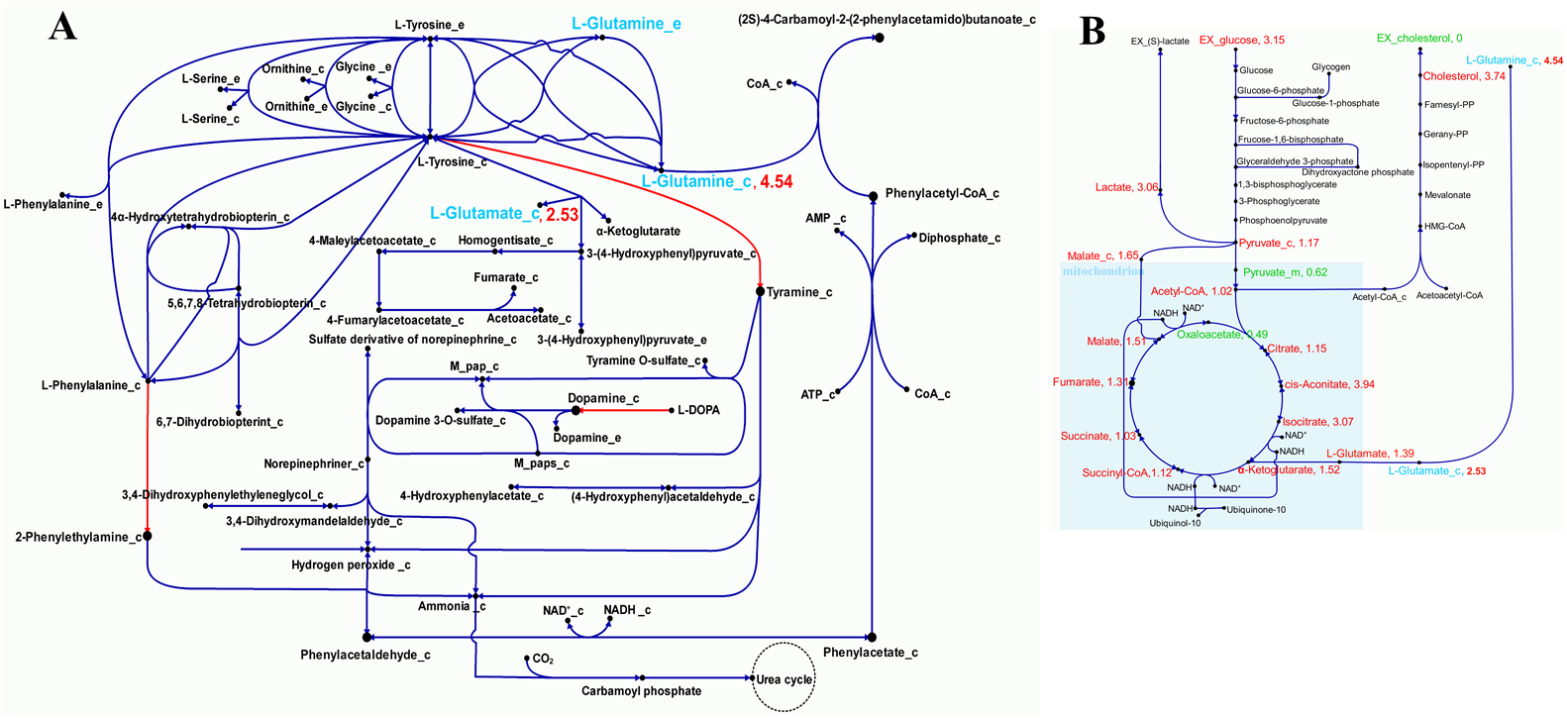
Metabolic reprogramming triggered by overexpression of *Ddc*. (A) Based on Recon2-hepatocyte model, three enzymatic reactions (red lines) are catalyzed by overexpression of Ddc. As a result of metabolic reprogramming, the fold change (FC) of the concentration of glutamine and glutamate are increased by 4.54 and 2.53, respectively (shown in blue). (B) Elevated expression of Ddc shift liver metabolism toward glycolysis and lactate synthesis in agreement with the hypothesis of the Warburg effect. The (FC) of metabolites in glycolysis, TCA cycle, and glutamine metabolism pathways are indicated in green (decrease) and red (increase).

As stated in the hypothesis of the Warburg effect, the production rates of metabolites in glycolysis, the tricarboxylic acid (TCA) cycle, and glutamine metabolism pathways can be altered by overexpression of a gene [1–8]. In this study, when DDC was fully overexpressed (100%), glucose uptake from the extracellular matrix and cellular pyruvate levels increased by fold changes (FC) of 3.15 and 1.17, respectively (Fig 3). Most pyruvate was further converted into lactate (3.06-FC) instead of being transported into the mitochondria (0.62-FC). Elevated DDC expression shifted liver metabolism toward glycolysis and lactate synthesis, which is in good agreement with the Warburg hypothesis (aerobic glycolysis). Contrary to the Warburg effect, we detected a slightly increased production rate (1.02-FC) of acetyl-coenzyme A (acetyl-CoA) in the mitochondria. The acetyl-CoA levels in the mitochondria were not affected possibly because of the conversion of other metabolites such as ketone bodies. Decreased oxaloacetate production (0.49-FC) in mitochondria can potentially impair the TCA cycle and led to mitochondrial respiratory defects.

In contrast, glutamine transported from the extracellular matrix was increased by 4.54-FC. Higher levels of glutamine were converted to glutamate (1.39-FC) by glutaminase. This reaction occurs in tumor cells [23] and was detected in the experimental metabolomic profiling (S5 Table). Glutamate was oxidized to α-ketoglutarate (1.52-FC), which then entered the TCA cycle generating higher citrate (1.15-FC), isocitrate (3.07-FC), and succinyl-CoA (1.12-FC) levels and eventually, higher ATP levels. Such metabolic reprogramming indicates that the glutaminolysis pathway serves as an alternative pathway to compensate for the production of cellular ATP. Despite decreased production of oxaloacetate (0.49-FC), which is catalyzed by malate dehydrogenase, the malate level in mitochondria still increased by 1.51-FC. This result implies that malate can be converted to pyruvate by the malic enzyme, which has been confirmed to play a crucial role in glutamine metabolism in rapidly growing tissues and tumors [40–42]. Lipid metabolism plays a critical factor of metabolic reprogramming in tumorigenesis [23]. We observed an apparent increase in the intracellular cholesterol level and a decreased extracellular cholesterol level when *Ddc* was overexpressed. The computational prediction was consistent with the experimental result as observed from Tsai et al. [23].

### BTK inhibitor and withaferin-A can reduce the protein level of DDC

**Fig 4:**
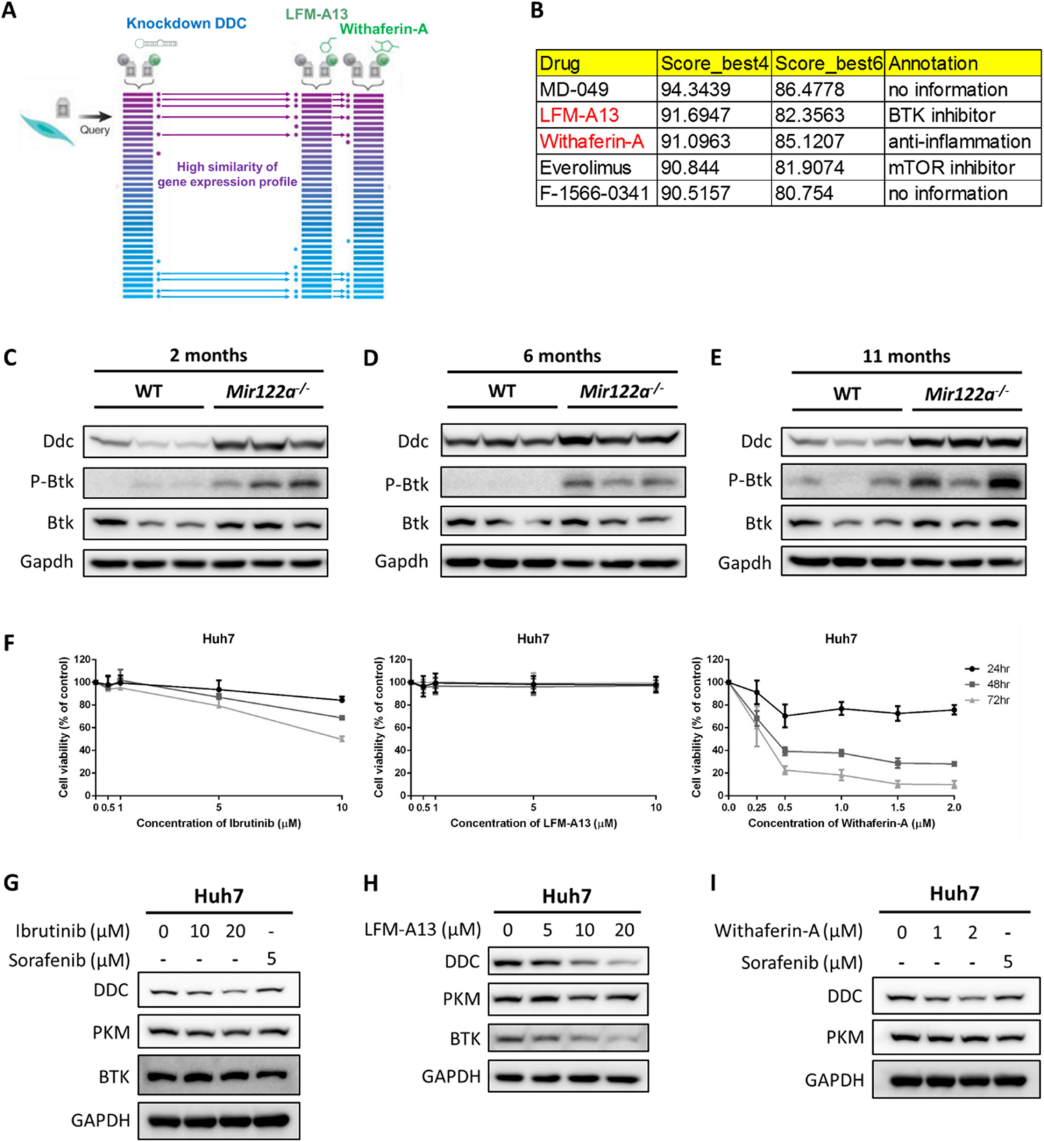
Downregulation of DDC expression by treatment with ibrutinib, LFM-A13, and withaferin-A. (A) Schematic illustration of similar gene expression signatures between *DDC* shRNA and chemical compounds. (B) We queried the *DDC* shRNA gene signature via LINCS database and found BTK inhibitor (LFM-A13), withaferin A, and several compounds shared similar gene expression profiles with DDC shRNA gene signature. Score_best4 and score_best6 are the mean connectivity scores across the four and six cell lines, respectively, in which the perturbagen connected most strongly to the query (*DDC* shRNA). (C, D, E) DDC, P-BTK, and BTK were upregulated in liver tissues from mir-122 knockout mice. (F) Huh7 cells were treated with various concentrations of ibrutinib, LFM-A13, and withaferin-A for 24–72 hours, respectively. Huh7 cells were treated with (G) 10 and 20 μM ibrutinib or 5 μM sorafenib, (H) 5–20 μM LFM-A13, and (I) 1 and 2 μM withaferin A or 5 μM sorafenib for 24 hours. Cell lysates were subjected to western blot analysis. DDC was downregulated by the treatment of 20 μM ibrutinib and 2 μM withaferin A, but not the treatment of sorafenib. Both DDC and BTK were downregulated by the treatment of LFM-A13 in Huh7 cells.

Since miR-122 knockout mice have increased levels of DDC (Fig 4C–4E), we then set up to determine the association between *DDC* and liver cancer. We used the database (PORGgeneV2, http://watson.compbio.iupui.edu/chirayu/proggene/database/index.php) [43] to search for *DDC* in the liver cancer dataset and then performed the survival analysis. From the dataset of GSE10141 [43, 44], high expression of *DDC* is associated with a poorer prognosis for patients with liver cancer (S4 Fig). A rational approach is to knock down *DDC* and then to perform microarray profiling of the knockdown cells to delineate the DDC-elicited signaling pathways. Alternatively, we can hypothesize that *DDC* knockdown cells may share similar gene expression patterns that result from certain drugs (Fig 4A), which may potentially modulate DDC-elicited signaling. Thus, we accessed the Library of Integrated Network-based Cellular Signatures (LINCS) (http://systemsbiology.columbia.edu/lincs), which contains 1.3 million L1000 microarray dataset of perturbational profiles spanning chemical compounds and gene knockdowns across multiple cell types. More importantly, LINCS provides a query interface to make inferences on the connections between the queries (e.g. *DDC* shRNA) and the internal (e.g. chemical compounds) gene expression profiles. We found that the gene expression profiles of several compounds had similar gene expression profiles with *DDC* shRNA. Of particular interest, withaferin-A and BTK inhibitor (LFM-A13), which share 91% and 92% identity, respectively, with the *DDC* knockdown gene expression profile (Fig 4B). LFM-A13, the BTK inhibitor in LINCS, is still in the pre-clinical development stage. Since the first FDA-approved BTK inhibitor is ibrutinib, we have also included ibrutinib in our assay. We examined the expression pattern of DDC in mir-122 knockout mice livers to explore the role of DDC. The results show upregulated profiles from 2, 6, and 11 months of mir-122 knockout mice liver data (Fig 4C–4E). In addition, we also examined the role of BTK in the mir-122 knockout mice livers. Interestingly, P-BTK and BTK were upregulated in mir-122 knockout mice livers as early as 2 months of age. Moreover, LFM-A13 and ibrutinib are BTK inhibitors. Together, BTK inhibitors may be potential drugs for liver cancer therapy. We next first determined the IC_50_ values of two BTK inhibitors (LFM-A13 and ibrutinib) in Huh7 cells to test whether these compounds could modulate DDC expression level. The IC_50_ values of both drugs were >10 μM in Huh7 cells (Fig 4F), whereas the IC_50_ of withaferin A in Huh7 cells was >2 μM (Fig 4F). Treatment of Huh7 cells with LFM-A13, ibrutinib, and withaferin A, but not sorafenib, which is the only FDA-approved drug for advanced HCC, could indeed result in downregulation of the protein expression level of DDC based on western blot analysis (Fig 4G–4I).

## Discussion

The Warburg effect, commonly observed in the metabolic reprogramming of cancer cells, is characterized by increased rate of glucose utilization and accumulation of lactate [45]. The findings of this study, which used 2-month-old *Mir122a* knockout mice for the modeling of metabolic reprogramming, addressed the disturbances in glucose utilization and accompanying pyruvate, lactate, and alanine metabolism at the pre-cancer stage (Fig 3). All the mice used in this work were male mice of 2-month old. It is an expansion of consideration of the (so-called) aerobic glycolysis and functional mitochondrial metabolism in supporting the energy-demanding biosynthetic pathways in cancer cells. Beyond that, this study also demonstrated that combined contribution of pyruvate and glutamine/glutarate/α–ketoglutarate was adapted and re-coordinated to drive TCA cycle for limited energy production (including ATP and redox coenzymes formation) (Fig 3 and S3 Fig). In this metabolic reprogramming, biosynthetic pathways, such as cholesterol biosynthesis for tumor cell growth, were minimally retained.

We selected the 20 genes listed in Fig 2 (also in S4 Table) to investigate other miRNAs that may co-regulate those genes. First, we identified experimentally verified miRNA-target pairs from the miRTarBase dataset (http://mirtarbase.mbc.nctu.edu.tw) and the starBase v2.0 (http://starbase.sysu.edu.cn/). Second, miRNA expression profiling of wild-type and *Mir122a*^*–/–*^mouse livers (both 2-months old) was performed using Small RNA sequencing [46]. We were able to identify miRNAs targeting 11 of the 20 genes with a total of 189 miRNA-target gene pairs. These genes are Acer2, Cpox, Fech, Gys1, Pafah1b1, Pank3, Pfkp, Rpia, Scd2, Sgpl1, Sptlc1 and Txnrd1. Differentially expressed miRNAs (expression ratio between *Mir122a*^*–/–*^and WT: 0.6≦KO/WT≧1.5) were found only in 40/189 pairs (S6 Table). Most of the miRNAs in *Mir122a*^*–/–*^are expressed at low (RPM<10) to moderate (RPM 10–100) levels compared to high level of miR-122-5p in normal mouse liver (RPM 21378.2). Since miR-122 is a highly abundant liver-specific miRNAs, an imbalance of the miRNA homeostasis in *Mir122a*^*–/–*^liver is anticipated. In addition, multiplicity of miRNAs targeting one gene is well noted. Whether those low-to-moderate levels of miRNAs impacted on the gene expression is difficult to evaluate. Clearly our results favor the notion that miR-122 plays a major dominant role in regulating these target genes in normal liver.

*Ddc*, *PKM*, and *Urod* exhibited the top three similarity ratios in Fig 2. *PKM* encodes to pyruvate kinase which catalyzes the transfer of a phosphoryl group from phosphoenolpyruvate to ADP, generating ATP and pyruvate. This kinase exhibited the second high similarity ratio (0.857) to Warburg hypothesis (metabolic reprogramming triggered by overexpression of *Pkm* was shown in S3 Fig); this finding indicates that production rates for pyruvate and oxaloacetate in the mitochondria are consistent with the hypothesis. This enzyme exhibited a similarity ratio of 0.556 to the metabolic data. *Urod* provides instructions for the formation of uroporphyrinogen decarboxylase, which is involved in the production of heme. Heme is vital for all organs in the body, and it is most abundant in the blood, bone marrow, and liver. Heme is an essential component of iron-containing proteins called hemoproteins, including hemoglobin. From this computation, this enzyme achieved the second highest score (0.694) of the LC/MS experiment but exhibited the third highest ratio (0.810) of the Warburg effect. Furthermore, the computational prediction indicated that the secretion flux for cholesterol in the extracellular space to the extracellular matrix was also reduced due to overexpression of UROD and PKM, respectively.

Four target genes in Fig 2 exhibited an infeasible solution for the computation when the genes were fully overexpressed. We were unable to attain a feasible solution for *ALDOA* if it was greater than 54% overexpressed to upregulate the corresponding reactions catalyzed by fructose bisphosphate aldolase. Its similarity ratio was 0.714 and 0.528 for the Warburg hypothesis and the experimental observation, respectively, with 54% overexpression. *Pfkp* encoded phosphofructokinase which catalyzes a rate-limiting step in glycolysis. It is highly regulated by small molecules for the promotion of glucose utilization for energy production, termination of glycolysis for gluconeogenesis initiation, or shunting hexoses into the pentose phosphate pathway. Phosphofructokinase should be downregulated due to the deficiency of miR-122a; otherwise, we could not obtain a feasible solution if it were assigned as upregulated. Its similarity ratio was 0.810 and 0.583 for the Warburg effect and the experimental observation, respectively, when *Pfkp* was 100% downregulated. *Cpt1a* could not up- or downregulate forward or backward reactions because it encodes as carnitine palmitoyltransferase 1A, which is present in the liver, to manipulate 85 fat acid oxidation reactions in the model. The Cpt1A enzyme is essential for fatty acid oxidation, a multistep process that breaks down fats and converts them into energy. Fatty acid oxidation takes place within mitochondria, which are the energy-producing centers in cells. *Cpt1a* needs to maintain oxidation-reduction at an equilibrium state if the forward reactions are upregulated, and its corresponding backward reactions should be upregulated simultaneously to maintain an equilibrium level. Here, *Cpt1a* was knocked out with fluxes of zero. Consequently, its similarity ratio was 0.714 and 0.583 for the Warburg effect and the experimental observation, respectively. *Pank3* encodes a protein belonging to the pantothenate kinase family, which is highly expressed in liver and catalyzes the first committed step and is the rate-controlling enzyme in CoA biosynthesis in bacteria and mammals. Pantothenate is the essential precursor for CoA, which is a cofactor for a multitude of metabolic reactions including the oxidation of fatty acids, carbohydrates, pyruvate, lactate, ketone bodies, and amino acids. According to our computation, the regulatory action of *Pank3* is similar to that of *Cpt1a*, which regulates the forward and backward reactions simultaneously to maintain CoA synthesis and catabolism in equilibrium.

In this study, 21 metabolites, consisted of TCA cycle and glutamine metabolism pathways, were used to evaluate the Warburg effects (S5 Table). Eighteen of these metabolites were obtained from hypotheses in the literature [1–8]. From the LC/MS experiments, three experimental metabolites, glutamine, glutamate, and alanine, were increased and consistent with Warburg hypothesis. From the computational prediction, glutamine transported from the extracellular matrix was increased (4.45-FC). A high level of glutamine was converted to glutamate by glutaminase. In addition, the increase of lactate and alanine was consistent with the results obtained from Hu et al. [47] that increased conversion of pyruvate to lactate and alanine by using hyperpolarized ^13^C-pyruvate in *Myc*-driven mouse liver cancer model. Furthermore, Pavlova and Thompson [48] described cancer-associated metabolic changes into six hallmarks. The article indicated that the import of an essential amino acid, leucine, through the plasma membrane localized neutral amino acid antiporter LAT1, which is coupled to a simultaneous efflux of glutamine [49]. In such a manner, intracellular glutamine may facilitate the import of a broad range of LAT1 substrates, including leucine, isoleucine, valine, methionine, tyrosine, tryptophan, and phenylalanine [50]. We found that five amino acids, such as leucine, valine, methionine, tryptophan, and phenylalanine contained in the experimental metabolomics profiling data increased and were consistent with the model predicted.

Using the online bioinformatics tool, LINCS, the gene expression profiles of withaferin A and BTK inhibitor share-gene expression profiles similar to the *DDC* knockdown profiles. Since LINCS has been terminated recently, similar result can be found in the new version of LINCS, CLUE (https://clue.io/). This analysis provides an opportunity to employ these available drugs (or drug repurposing) to target DDC expression. In fact, empirical evidence also suggests that withaferin A and BTK inhibitors can downregulate DDC, but not PKM2, expressions (Fig 4). BTK inhibitors have emerged as crucial therapeutic agents for distinct cancer treatment. The first FDA-approved BTK inhibitor is ibrutinib. The indication for ibrutinib is for the treatment of patients with Mantle cell lymphoma, chronic lymphocytic leukemia, and one kind of non-Hodgkin’s lymphoma (Waldenström’s macroglobulinemia). Moreover, there are still many innovative compounds in preclinical or clinical development, including GDC-0834, CGI-560, CGI-1746, HM-71224, CC-292, ONO-4059, and CNX-774 [51], raising the possibility that some of these compounds might have effects similar to ibrutinib. Withaferin A has many functions, including antioxidative, anti-inflammatory, antiproliferative and apoptosis-inducing properties. Recently, many studies show that withaferin-A can reduce the growth of multiple tumor types in the mouse model [52]. To further elucidate the role of DDC, we knocked out DDC in Huh7 cell line via CRISPR/Cas9 system and then performed microarray profiling of the knockout cells. The differentially expressed gene signatures were used to query the ConsensusPathDB (http://consensuspathdb.org/) [53] database to search for DDC-elicited signaling pathways. The metabolism pathway received the highest score than other pathways (manuscript in preparation). Our data further extend the potential use of these drugs as metabolism regulators in liver cancer cells.

One metabolic profiling dataset for human HCC [54] was also compared with the computational prediction of the Recon 2-hepatocyte model. According to the U.S. National Library of Medicine, the secretion rates of direct bilirubin and indirect bilirubin in humans are restricted by 0 ≤ *v*_direct_bilirubin_≤ 5.13 mol/L/day and 5.13 ≤ *v*_indirect_bilirubin_≤ 27.36 mol/L/day, respectively. We applied 560 gene-encoded enzymes listed in the model to compute the flux distributions in the normal and cancer states. From the computation, 13 gene-encoded enzymes exhibited high similarity ratios to the Warburg effect (S7 Table), and are shared by the *Mir122a*^–/–^mouse case and human HCC case. Four of them, *DDC*, *PKM*, *ENTPD4*, and *ALDOA*, are miR-122 target genes. *DDC* scored the highest similarity ratio (0.905) to the Warburg effect and the experimental metabolomics profiling data (0.513) in human HCC case (S7 Table). The metabolic reprogramming from the computation could be applied to predict the oncogenic behavior.

A genome-scale human metabolic model can be applied to identify disease genes and disease pathways, offering more appropriate targets, such as DDC, for drug development. MicroRNAs mediate fine tuning of the genes that are directly or indirectly involved in cancer metabolism. In summary, this study integrated the genome-scale metabolic model of hepatocyte, namely Recon 2-hepatocye model, and experimental data of 2-month old *Mir122a*^–/–^mice to infer the metabolic reprogramming that was initiated in the early stages of cancer development. DDC has not only exhibited the highest similarity ratios to the biological hypothesis of Warburg effect and the experimental observation in *Mir122a*^–/–^mice, but also demonstrated its importance in human HCC cases. Moreover, the genome-scale metabolic model could be applied to rationally analyze flux distributions for the normal and dysregulated cell. Finally, the role of ibrutinib on metabolism in cancer warrants further investigation.

## Materials and methods

The wild type and *Mir122a*^–/–^mice were housed in the Animal Center of National Yang Ming University and were handled following institutional guidelines. The mice were fed with the Laboratory Autoclavable Rodent Diet 5010, (http://www.labdiet.com/) which contains 69 chemical compounds in the ingredients. Twenty compounds, as listed in S8 Table, are represented as the metabolites in the Recon 2-hepatocye model. On the basis of the feeding instructions for Rodent Diet 5010, we set the molar fraction of each compound to the upper bound of the uptake rate while analyzing the flux distributions in normal and *Mir122a*^*–/–*^mice.

This study employed a liver-specific metabolic network model reconstructed from Recon 2, which is the second version of the largest reconstruction of the human genome [10]. It consists of 2163 metabolites and 3047 reactions in eight compartments and was used to predict the metabolic capability under a particular condition and to infer the metabolic reprogramming of hepatocytes under miR-122 dysregulation.

### Ethics statement

The animal were euthanized by CO_2_ following the institutional guidelines. All the studies were conducted in accordance with the Guidelines for the Care and Use of Mammals in Neuroscience and Behavioral Research and were approved by Institutional Animal Care and Use Committee (IACUC) of National Yang Ming University.

### Computational methods

FBA is an *in silico* flux-based optimization model for predicting the metabolic flux distributions in genome-scale metabolic networks. Such an optimization problem usually includes a cellular objective, (*e*.*g*., maximization of cell growth). The biomass reaction actually contains in Recon 2-hepatocyte model, but it is a block reaction in the model. We therefore considered FBA with the maximization of ATP production as an objective in this study.

{maxv(cfTvf+cbTvb)subjecttoN(vf−vb)=0vi=viReg,i∈ΩRegvjLB≤vj≤vjUB,j∉ΩReg (1)

where *v*_*f*_ and *v*_*b*_ are the irreversible forward and backward fluxes, respectively, for the production of a metabolite such as ATP, **N** is an *m*×*n* stoichiometric matrix where *m* is the number of metabolites and *n* is the number of reactions, *v*_*j*_^*LB*^ and *v*_*j*_^*UB*^ are the positive lower and upper bounds of the *j*^th^ flux, respectively, and *v*_*i*_^Reg^ is the *i*^*th*^ up- or downregulated flux due to the *i*^*th*^ enzyme dysregulation. The value for the forward or backward flux is computed by the following equations:

$$\begin{array}{l} \mathrm{Up-regulation}\;\mathrm{for}\;v_{i,f}\;\mathrm{or}\;v_{i,b}:\\ \left\{ \begin{array}{l} v_{i,b/f}=v_{i,b/f}^{basal}\\ v_{i,f/b}=v_{i,f/b}^{basal}+\delta \left(v_{i,f/b}^{\max}-v_{i,f/b}^{basal}\right) \end{array}\right. \end{array}$$ (2)

$$\begin{array}{l} \mathrm{Down-regulation}\;\mathrm{for}\;v_{i,f}\;\mathrm{or}\;v_{i,b}:\\ \left\{ \begin{array}{l} v_{i,b/f}=v_{i,b/f}^{basal}\\ v_{i,f/b}=v_{i,f/b}^{basal}+\delta \left(v_{i,f/b}^{\min}-v_{i,f/b}^{basal}\right) \end{array}\right. \end{array}$$ (3)

where *v*_*i*_^*basal*^ is the basal flux, *v*_*i*_^max^ and *v*_*i*_^min^ are the maximum and minimum fluxes, respectively, at a normal state, and *δ* is the regulation strength parameter between 0 and 1.

The optimal flux distribution of the flux-balance problem expressed by Eq (1) is not unique, and there is a large set of alternative flux distributions with identical values for the objective function. We minimized the squared sum of all internal fluxes for FBA to ensure efficient channeling of all the fluxes through all pathways to eliminate the multiplicity of flux values due to the problem expressed in Eq (1). The second optimization problem can be reformulated as the principle of flux minimization [55] if the equilibrium constant for each reaction is available. Such a problem is to using minimum enzyme activities to enhance cellular capacity. The minimizing Euclidean norm problem is expressed as:

{minv∑i∈ΩInt(vf,k)2+(vb,k)2subjecttoN(vf−vb)=0vi=viReg,i∈ΩRegvjLB≤vj≤vjUB,j∉ΩRegcfTvf+cbTvb≥z* (4)

where z* is the maximum specific metabolite production rate obtained from the problem expressed in Eq (1). The problem expressed in Eq (4) is a quadratic programming problem that can numerically achieve a unique solution.

Flux balance equations in (1) and (4) omit the dilution term during cell growth. The dilution rate should be included in the mass balance equations of the intracellular metabolites to cope with cell growth [56, 57]. The specific growth rate is generally in terms of a kinetic constant and concentration of uptake nutrients. Metabolite dilution flux balance analysis (MD-FBA) [58] and flux imbalance analysis [59] are applied to surmount such a weakness to predict flux distribution in genome-scale metabolic networks. Both methods can be applied to this study in order to improve predictability of flux distributions. However, the cell-growth rate in Recon 2-hepatocyte model is a block reaction so that the flux value is zero [10]. In the future, a liver-specific metabolic model has to be reconstructed to achieve a non-zero flux of cell growth rate to account for metabolite dilution.

In the computational viewpoint, the production rate of a metabolite is involved in different compartments, and it can be calculated through a GSMM. However, this work applied an LC/MS experiment to obtain the metabolomic profiling for the normal and dysregulated cell. According to the protocol of LC/MS experiment, the liver tissues were minced in small chunks and were rapidly frozen in liquid nitrogen, and then the tissue was homogenized to prepare a sample to determine the metabolomic profiling. Such a homogenization was indicated that metabolites contained in different compartments was destroyed. As a result, LC/MC was applied to measure the homogenized metabolite concentrations. In order to compare with LC/MS experiments, the metabolite involved in different compartments has to sum up to yield the overall production rate of the metabolite which is an intracellular compound of the cell. The overall production rate (*r*_*m*_) of each intracellular compound at deficient and normal states is respectively evaluated as

$$r_{m}=\sum_{i\in \Omega^{c}}\left(\sum_{N_{ij}>0,j}N_{ij}v_{f,j}-\sum_{N_{ij}<0,j}N_{ij}v_{b,j}\right),m\in \Omega^{m}$$ (5)

where *N*_*ij*_ is the stoichiometric coefficient for the *i*^*th*^ metabolite participating in the *j*^*th*^ reaction. An intracellular compound (or metabolite) exists in different compartments of the metabolic network; therefore, the rates for all compartments, Ω^c^, are summed to provide the production rate of the metabolite. The production rate is then applied to compute the fold change (FC) at deficient and normal states and to evaluate the similarity ratio between the computational results and experimental observations. The logarithmic FC (*LFC*_*m*_) for the *m*^*th*^ metabolite and similarity ratio (*SR*) are respectively expressed as

$$LFC_{m}=\log_{2}\left(FC_{m}\right)=\log_{2}\left(\frac{r_{m,\mathrm{deficient}}}{r_{m,\mathrm{normal}}}\right)$$ (6)

$$SR=\frac{\sum_{m=1}^{N_{data}}\mu_{m}}{N_{data}}$$ (7)

where the similarity indicator (μ_*m*_) for each metabolite is defined as:

$$\mu_{m}=\left\{ \begin{array}{l} 1,\;\mathrm{sgn}\left(LFC_{m}\right)=\mathrm{sgn}\left(LFC_{m}^{Exp}\right)\\ 0,\;\mathrm{otherwise} \end{array}\right.$$ (8)

where sgn is defined as the signum function. The similarity indicator is a qualitative comparison that we hypothesize the prediction is similar to the experiment if an increase/decrease of the overall production rates between deficient and normal states is consistence with the change for experimental results.

The production rate of a metabolite obtained from the computation is not equal to the concentration of that metabolite achieved through LC/MS experiments. Rates of metabolites are in term of their regulated enzyme activities and metabolite concentrations. Such kinetic models can be used to predict concentrations if model parameters are given in advance. A genome-scale kinetic model is generally not available up to date. As a result, the constraint-based model can be applied to predict fluxes in the genome-scale metabolic network. In contrast to kinetic models of metabolism, a shortage of constraint-based approaches is the incapacity to predict metabolite concentrations, but it can be used to analyze the genome-wide flux distribution. In this study, we firstly compute the metabolite flux-sum distributions in the normal and deficient states, respectively. The flux-sum changes between these states are then compared with the changes of LC/MS metabolomics observations in the normal and cancer states. The similarity indicator in Eq (8) is used as a measure to inspect whether the trend of flux change is coincided with the trend of experimental observations and Warburg hypothesis, i.e. the similarity indicator is assigned to be one if the flux change in the normal and deficient states is similar to concentration change (A toy example shown in S1 Table). The similarity ratio denotes the percentages of the computational predictions are similar to the experimental observations. As a result, the similarity ratio is used as an measure to explain variation of overall fluxes based on metabolite-centric approach alter from the normal state to the deficient situation.

This study was not only used LC/MS metabolomic data of mice lacking *Mir122a* for evaluating the similarity ratio, Warburg hypothesis was also applied to compute the similarity ratio. Indeed, the Warburg effect accessed from literatures [1–8] hypothesizes that it could trigger the production rate of metabolite to be increase/decrease, not concentration. In this study, 21 metabolites, consisted of TCA cycle and glutamine metabolism pathways, were used to evaluate the Warburg effects (S5 Table). We found three metabolites, glutamine, glutamate, and alanine, were also contained in LC/MS experiments, and their concentrations were increased and similar trend as Warburg hypothesis. Furthermore, Pavlova and Thompson [47] have recently reviewed cancer-associated metabolic changes to enhance the import of an essential amino acid, leucine, through the plasma membrane localized neutral amino acid antiporter LAT1, which is coupled to a simultaneous efflux of glutamine. In such a manner, intracellular glutamine may facilitate the import of a broad range of LAT1 substrates, including leucine, isoleucine, valine, methionine, tyrosine, tryptophan, and phenylalanine. We found that five amino acids, i.e. leucine, valine, methionine, tryptophan, and phenylalanine contained in the mouse experimental metabolomics profiling data increased and were similar to the increasing fluxes of the hypothesis and model prediction. We additionally acquired metabolomics profiling data for human HCC [53], and found that the trend of metabolic alternation was similar to Warburg hypothesis (see S7 Table). All optimization problems were solved using the CPLEX solver accessed from GAMS on a 3.4 GHz Intel Core i7 CPU with 32 GB of RAM. The source code is available in the supporting information (S1 File).

### Metabolomic analysis by liquid chromatography–mass spectrometry

A modified Folch’s method was used for hydrophilic and hydrophobic metabolite extraction [60]. Approximately 0.3 g of frozen liver tissue was homogenized in liquid nitrogen and transferred to a 20-mL glass tube. Subsequently, 6 mL of chloroform/methanol (2:1, v/v) solution and 1.5 mL of water were added. The mixture was vortexed four times for 30 s and subsequently centrifuged at 700 × *g* for 30 min at 4°C. The upper phases (hydrophilic phase and water soluble phase) were transferred to new glass tubes and then dried under a stream of nitrogen. The residues were collected and stored at −80°C. The residues were suspended in 100 μL of 95:5 water/acetonitrile and centrifuged at 14,000 × *g* for 5 min. The clear supernatant was collected for liquid chromatography–mass spectrometry (LC/MS) analysis.

Liquid chromatographic separation was achieved on a 100-mm×2.1 = mm Acquity 1.7-μm C8 column (Waters Corp., Milford, USA) using the ACQUITY UPLC system (Waters Corp., Milford, USA). The column was maintained at 45°C and a flow rate of 0.5 mL/min. Analytes were eluted from LC column using with a linear gradient: 0–1.25 min: 1%-50% B; 1.25–2.5 min: 50%-99% B; 2.5–5.0 min: 99% B; 5.1–6 min: 1% B for re-equilibration. The mobile phase was 0.1% formic acid in water (solvent A) and acetonitrile (solvent B).

The eluent was introduced into the Synapt G1 high-definition mass spectrometer (Waters Corp., Milford, USA) operated in the positive ion mode. It is a time of flight mass spectrometer (TOFMS) and this system is less than 5 ppm mass error in specification. The specification was checked in every study to make sure the mass accuracy. The following conditions were used: the desolvation gas was set to 700 l/h at a temperature of 300°C, the cone gas was set to 25 l/h, and the source temperature set at 80°C. The capillary voltage and cone voltage were set to 3,000 V and 35 V, respectively. The MCP detector voltage was set to 1,650 V. The data acquisition rate was set at 0.1 s with a 0.02 s interscan delay. The data were collected in centroid mode from 20 to 990 m/z. For the long-term study, all analyses of the LCTOFMS were acquired using the lock spray to ensure accuracy and reproducibility. For accurate mass acquisition, a lock-mass of sulfadimethoxine at a concentration of 60 ng/mL and a flow rate of 6 μL/min (an [M+H]s^+^ ion at 311.0814 Da in ESI positive mode) were used. The lock spray frequency was set at 10 s.

For the purpose of quality control (QC) in LC-MS performance was also prepared. As to the QC sample, 10 μl aliquot of supernatant that had been extracted of each sample was mixed and the LC-MS experiment was performed together with the samples and replicates from the same LC-MS condition. The QC sample was applied before and after injection of every 20 samples. Each sample was analyzed six replicates. We checked six replicates in the QC and each sample. Each sample including QC was shown highly reproducibility in principal component analysis (PCA) and orthogonal partial least square analysis (OPLS-DA) plots (S5 Fig).

Raw mass spectrometric data were processed using MassLynx V4.1 and MarkerLynx software (Waters Corp., Milford, USA). The intensity of each mass ion was normalized with respect to the total ion count to generate a data matrix including the retention time, m/z value, and the normalized peak area. The multivariate data matrix was analyzed by SIMCA-P software (version 13.0, Umetrics AB, Umea, Sweden). Orthogonal projection to latent structure-discriminant analysis (OPLS-DA) was carried out before the Pareto scaling was applied. This software had been used for multivariate data analysis and representation.

Precise molecular mass data for metabolites, which showed significant differences between two groups, were then submitted for database searching, either using either an in-house database or online HMDB (http://www.hmdb.ca/) or the METLIN (metlin.scripps.edu/index.php) database. For identifying specific metabolites, standards were subject to UPLC-MS/MS analyses under the conditions that were identical to those of the profiling experiment. MS/MS spectra were collected and confirmed by chemical standards or database match from HMDB or METLIN.

#### Statistical analyses

To maximize identification of differences in metabolic profiles between groups, an OPLS-DA model was applied and performed using the SIMCA-P software (version 13.0, Umetrics AB, Umea, Sweden). The variable importance in the projection (VIP) value of each variable in the model was calculated to indicate its contribution to the classification. A higher VIP value represents a stronger contribution to discrimination between the groups. The VIP values of those variables greater than 1.0 are considered significantly different. Results are expressed as the mean ± SD for continuous variables and as the number (percentage) for categorical variables. Data were compared by two-sample *t*-tests. A p value of < 0.05 was considered significant.

#### Metabolite set enrichment analysis

Among those significantly altered features of metabolomics profiling data, 35 were matched to the human metabolites database (HMDB). The mapped species were assigned an HMDB ID for subsequent MSEA analysis implemented in the MetaboAnalyst assessment [61].

### Cell culture

The Huh7 cell line was obtained from Dr. Zhong-Zhe Lin, National Taiwan University Hospital, Taiwan. Huh7 cells were cultured in DMEM and supplemented with 10% fetal bovine serum (FBS, Invitrogen), 10 U/ml penicillin, 1% nonessential amino acids and 2 mM L-glutamate in an incubator with 5% CO_2_ at 37°C.

### Western blotting

Cells (5 × 10^5^ cells) were seeded in 6-cm tissue culture dishes for overnight incubation and were then treated with ibrutinib (Santa Cruz Biotechnology, sc-483194), LFM-A13 (Enzo, BML-EI295), and withaferin A (Enzo, BML-CT104), respectively, for 24 hours. Mice liver tissues were harvested from C57BL/6 wildtype and mir-122 knockout mice at 2, 6 and 11 months of age. All samples were lysed in lysis buffer (25 mM Tris-HCl pH 7.6, 137 mM NaCl, 1 mM EDTA pH 8.0, 1 mM EGTA pH 8.0, 1% Triton X-100, 2 mM sodium pyrophosphate, 25 mM β–glycerol phosphate) supplemented with protease inhibitor and phosphatase inhibitor. All samples were denatured by heating at 95°C for 5 minutes. The total protein was electrophoresed on 10%, 12% SDS-polyacrylamide gel and transferred onto PVDF membranes (Millipore). The membrane was blocked with 5% non-fat milk at room temperature for 1 hour. The membrane was incubated with the primary antibody at 4°C overnight. They were washed with TBST three times (10 minutes per time). The membrane was incubated with the HRP-conjugated secondary antibody at room temperature for 1 hour. They were washed with TBST three times (10 minutes per time). The proteins were detected using an enhanced ECL.

## Supporting information

S1 Fig Metabolomics analyses of *miR-122a* deficient mice. (TIF)

S2 Fig Similarity ratio related to different regulation strength parameters for triggering overexpression of *Ddc*. (TIF)

S3 Fig Metabolic reprogramming triggered by overexpression of *Pkm*. (TIF)

S4 Fig High *DDC* mRNA expression is a negative prognostic factor in patients with hepatocellular carcinoma. (TIF)

S5 Fig Reproducibility in principal component analysis (PCA) and orthogonal partial least square analysis (OPLS-DA) plots. (TIF)

S1 Table The fold changes of 35 intracellular metabolites from experimental results and computational predictions of 20 miR122a target enzymes. (XLSX)

S2 Table miR122a target genes of mouse. (XLSX)

S3 Table miR122 target genes of human. (XLSX)

S4 Table Genes regulated by miR-122 and their regulated reactions in Recon2-hepatocyte model. (XLSX)

S5 Table Identification of significant metabolites between deficient and normal state from experimental results and the hypothesis of Warburg effect respectively. (XLSX)

S6 Table Differentially expressed miRNAs in the livers of 2-month-old male *Mir122a*^*–/–*^and WT mice. KO/WT, Expression ratio between *Mir122a*^*–/–*^and WT (0.68≦KO/WT≧1.5).
(XLSX)

S7 Table TOP 13 enzymes exhibited the higher similarity ratio to Warburg effect. (XLSX)

S8 Table Twenty compositions in the Recon2-hepatocyte model are set the molar fraction of each composition to the upper bound of the uptake rate. (XLSX)

S1 File Similarity file. GAMS file used for evaluating similarity ratio.
(ZIP)
