# Supplementary figures and images for: Flux balance analysis predicts Warburg-like effects of mouse hepatocyte deficient in miR-122a
Source: PLoS Comput Biol. 2017 Jul 7;13(7):e1005618. doi: 10.1371/journal.pcbi.1005618 (PMC5536358; doi:10.1371/journal.pcbi.1005618)

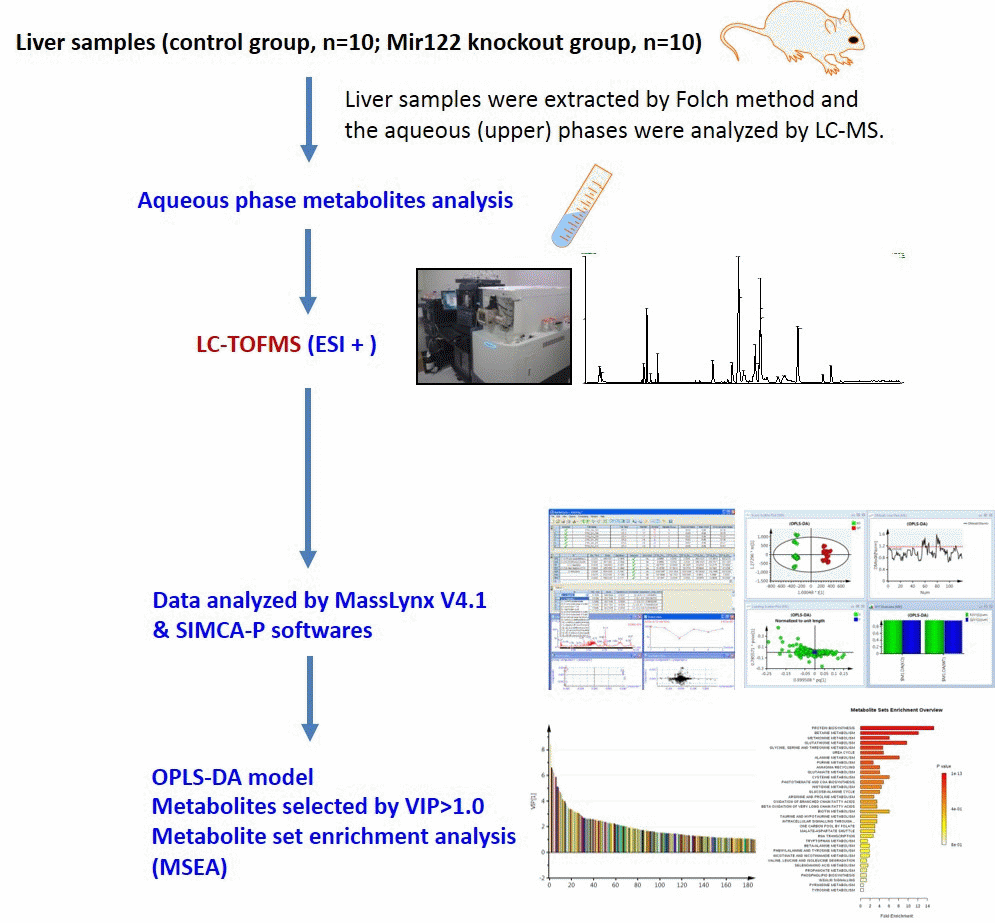

Supplement: S1 Fig — (TIF) [file pcbi.1005618.s001.tif]

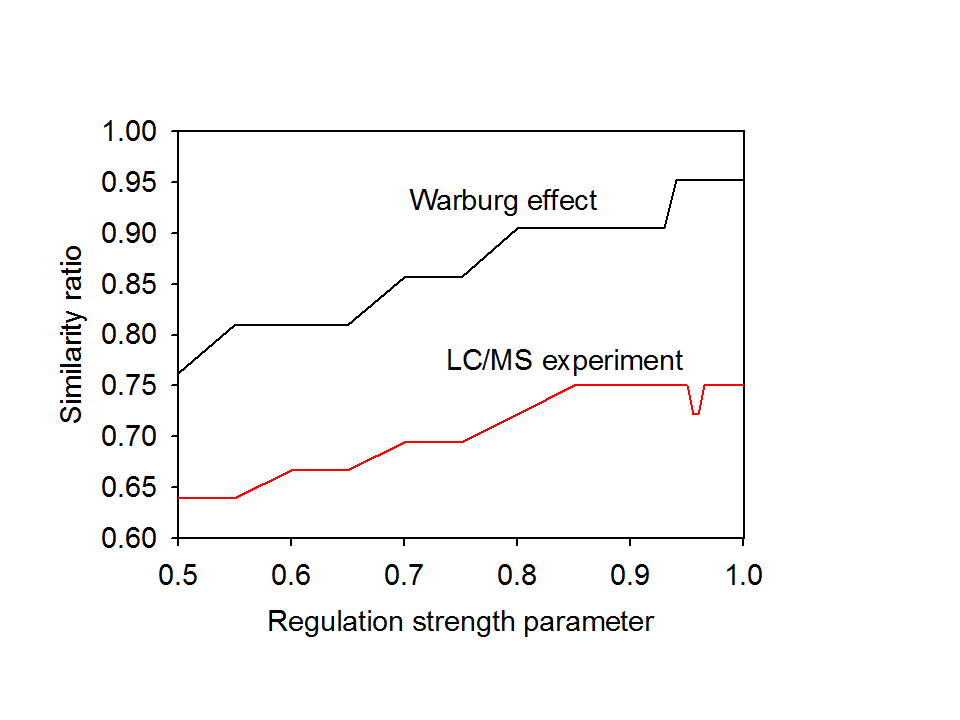

Supplement: S2 Fig — (TIF) [file pcbi.1005618.s002.TIF]

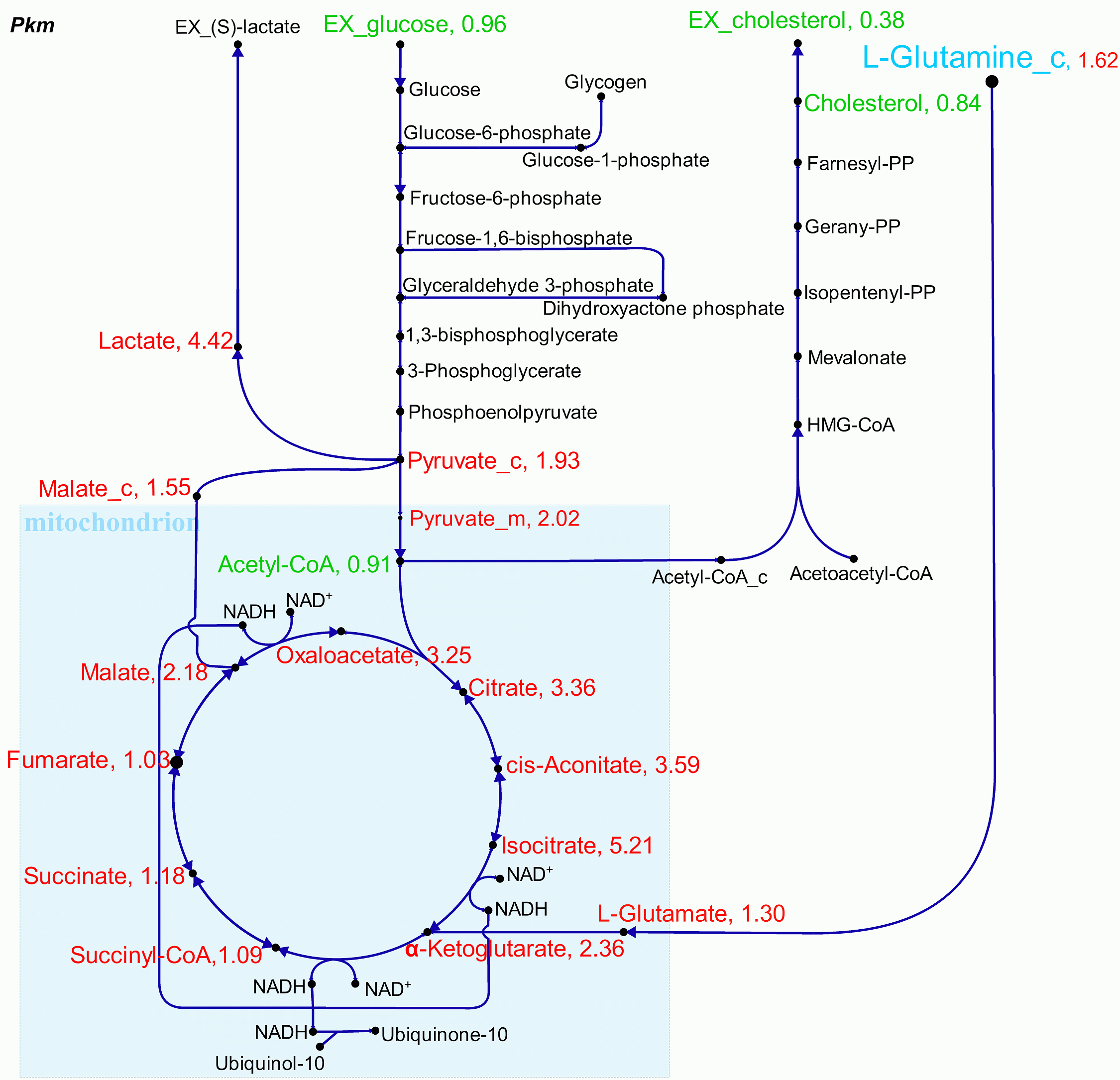

Supplement: S3 Fig — (TIF) [file pcbi.1005618.s003.tif]

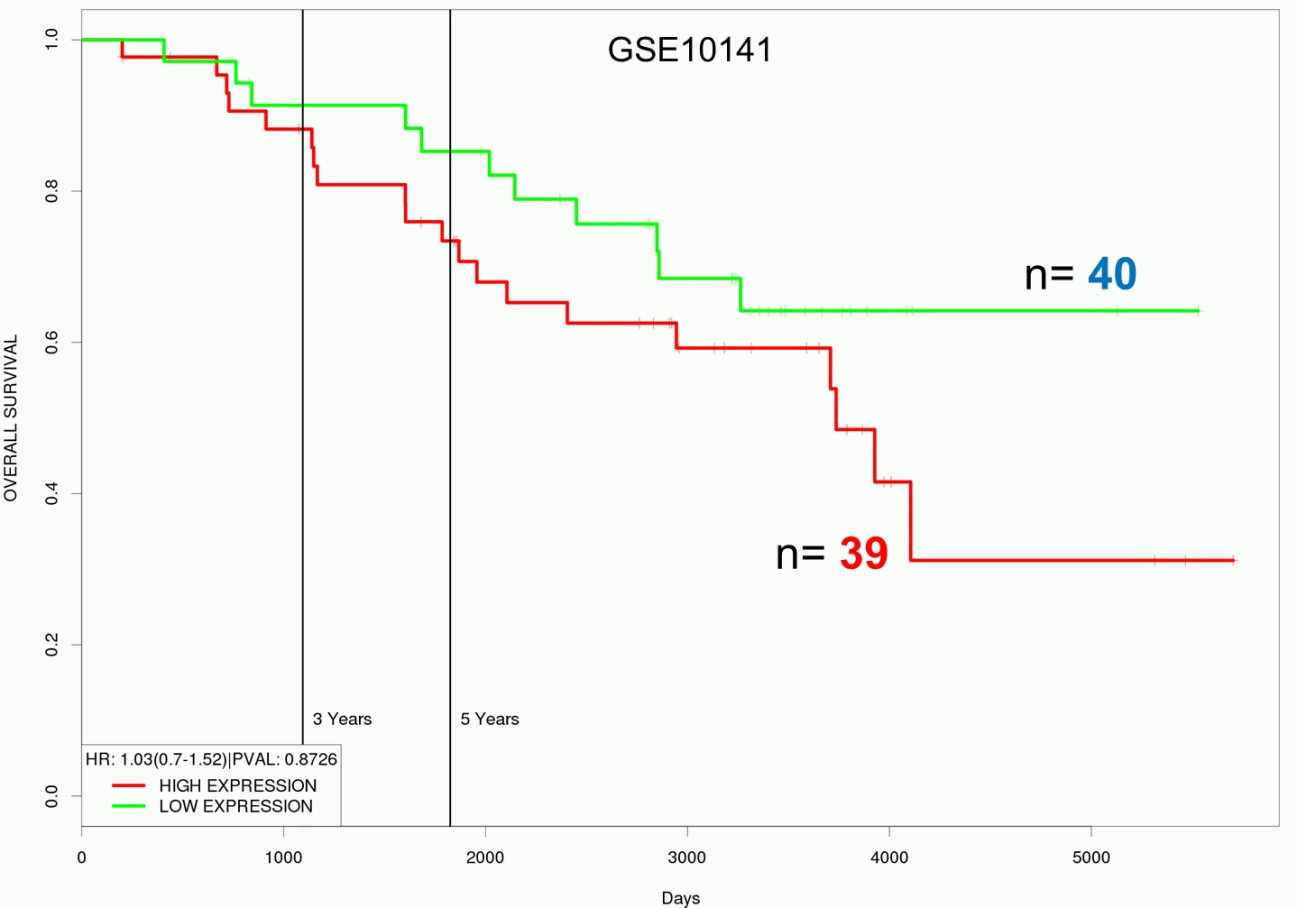

Supplement: S4 Fig — (TIF) [file pcbi.1005618.s004.tif]

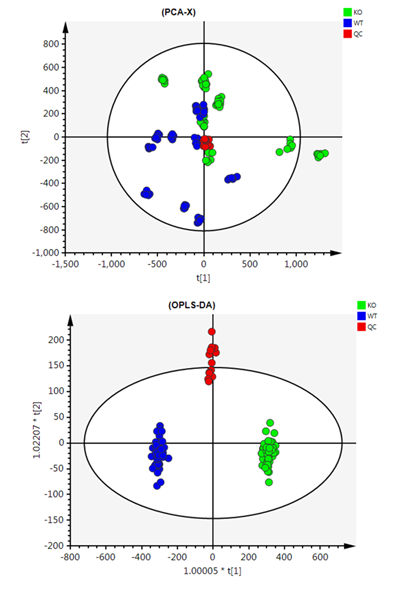

Supplement: S5 Fig — (TIF) [file pcbi.1005618.s005.tif]
